# Identifying Staff Behaviors and Client Outcomes of High-Performing Staff at a National Homeless Hotline for Veterans

**DOI:** 10.1007/s11414-026-09996-0

**Published:** 2026-04-27

**Authors:** Jack Tsai, Laura Witte, Aerin DeRussy, Amanda Burden, Ann Elizabeth Montgomery

**Affiliations:** 1 U.S. Department of Veterans Affairs, Homeless Programs Office, 810 Vermont Ave, NW, Washington, DC, 20420 USA; 2 Department of Management, Policy, and Community Health, University of Texas Health Science Center at Houston School of Public Health, Houston, TX USA; 3 Birmingham, AL VA Health Care System, Birmingham, AL USA; 4 U.S. Department of Veterans Affairs, National Call Center for Homeless Veterans, Washington, DC, USA; 5 Department of Health Behavior, University of Alabama at Birmingham School of Public Health, Birmingham, AL USA; 6 U.S. Department of Veterans Affairs, South Texas Veterans Health Care System, San Antonio, TX USA

## Abstract

Crisis call centers serve important functions for public health. With the rise of artificial intelligence (AI) and potential roles in crisis call centers, it is important to examine what human behaviors influence the effectiveness of call center outcomes. The authors studied the U.S. Department of Veterans Affairs (VA) National Call Center for Homeless Veterans (NCCHV) to (1) examine differences in staff behaviors between top and average performers and (2) evaluate client outcomes of top-performing and average-performing staff. Of 159 NCCHV staff, 10 top-performing and 10 average-performing staff were randomly selected based on their annual performance ratings from fiscal years 2020–2023. For each NCCHV staff selected, three audio-recorded calls were sampled and blindly rated by researchers on a list of nine coded behaviors (e.g., empathy, thoroughness). Healthcare outcomes of clients served by these NCCHV staff were examined through analysis of their VA medical records data. Results found that top-performing staff were significantly more likely to be rated as tactful and empathetic, thorough with information gathering, and less likely to be rated as providing inappropriate advice and being forceful/rigid with clients than average-performing staff. Clients served by top-performing NCCHV staff showed significant increases in VA healthcare use including outpatient healthcare and homeless services after calling NCCHV which were not observed in clients served by average-performing NCCHV staff. Together, these findings suggest human behaviors of homeless call center staff influence client engagement with care, emphasizing the importance of staff training and caveating any potential use of AI in these call centers.

## Introduction

Homelessness prevention is an essential, but understudied, part of public health efforts to end homelessness in communities across the USA. The U.S. Department of Veterans Affairs (VA) has led some of the largest efforts to address homelessness, including the creation of the National Call Center for Homeless Veterans (NCCHV) in December 2009. NCCHV is a 24 h/7 days-a-week call center that veterans, family members and friends, and VA and non-VA providers can access to connect veterans with VA and local community-based services.^1^ Contacts to NCCHV can be made by phone through NCCHV’s hotline or through an anonymous electronic chat function at the NCCHV website. Those who contact the NCCHV by chat can choose to be connected to a NCCHV staff member on a call line. NCCHV is staffed by trained agents, referred to as “responders,” who respond to contacts via telephone or chat and use standardized scripts to gather information about contacts and their needs, provide resources, and produce a consult (i.e., referral) to a local VA point of contact for follow-up.^2^ All veterans can access NCCHV, although their eligibility for VA care following their contact with NCCHV may depend on their military history, discharge status, and priority group.

Following recommendations from a 2014 report by the VA Office of Inspector General, NCCHV implemented performance metrics and established quality controls.^1^ An early study of NCCHV reported that 110,197 unique veterans had contacted the call center between 2018 and 2020.^3^ Nearly all contacts with NCCHV (90%) ended with a consult placed to the local VA homeless program point of contact. Among veterans who contacted NCCHV and had an existing VA medical record, large increases were observed in their use of VA homeless programs following contact. There were also small, significant increases in use of medical and mental health services and substance use disorder treatment after NCCHV contact. These findings indicate the important role NCCHV, and perhaps other similar call centers, may play in helping connect individuals to needed healthcare and social services.

More recent studies of NCCHV have provided further information. One study of 251,668 veterans who contacted NCCHV between 2019 and 2023 found that annual contacts to NCCHV increased over this 5-year period (from 79,802 contacts in 2019 to 184,504 contacts in 2024) demonstrating the demand for such services.^4^ This study also found that 24% of veterans who contacted NCCHV had not used any VA care in the 2 years prior to their contact, showing the potentially important role that NCCHV can play in facilitating engagement with care. The second study found that veterans who reported being currently homeless (instead of at-risk of homelessness) and were white, Asian, or other race, lived on the West Coast, or had an other-than-honorable discharge were less likely to engage with VA care after contacting NCCHV.^5^ Veterans who did not contact NCCHV themselves (i.e., somebody contacted NCCHV on their behalf) were also less likely to engage with VA care after NCCHV contact.

While there have been several decades of research on call centers related to suicide, domestic violence, and other crises,^6–8^ including the VA’s Veterans Crisis Line,^9^ studies on homeless-specific call centers have been very rare despite their existence in many large cities.^10^ An important emerging issue is the role of artificial intelligence (AI) in crisis call centers and whether AI agents may be able to play a role in handling calls. For example, AI-powered chatbots such as those based on large language models (LLMs) have been explored as replacements for suicide crisis hotline workers in the UK.^11^ There has also been interest in AI, not to replace human workers, but to assist in collecting information from callers and measure their distress to help human workers tailor services to their needs in Australia.^12^ As AI develops, there are many forms that AI may be integrated to assist crisis support services, from providing front-end interactions to analyzing data on the backend in real time. Some argue that AI can fulfill the needs of many call centers, while others point to important elements of human connection and behaviors that are needed to assist callers in crisis.^11,13,14^ More research is needed to understand the best ways to engage callers on homeless hotlines, what important human behaviors are essential, and ways to improve callers’ experiences during a critical period for homeless prevention.

The current study had two major aims: (1) identify unique behaviors between the 10 top-performing and 10 average-performing NCCHV responders and (2) examine VA healthcare engagement outcomes of clients served by top-performing and average-performing NCCHV responders. The results can not only inform NCCHV operations and other homeless call centers but contribute to needed knowledge about important staff behaviors in an era of growing AI use across service sectors.

## Methods

### NCCHV responders and contacts

NCCHV maintains a monthly performance report card for each NCCHV responder about elements of their performance. Responders receive scores across three critical elements—adherence to schedule, customer service, and quality monitoring reviews—which are based on performance metrics, call log records, and supervisor ratings. NCCHV responders receive annual performance reviews from their supervisors based in part on these monthly performance report cards; these reviews do not include metrics related to responders linking clients with services. Annual performance reviews are rated by supervisors on the following scale: 1 = unsatisfactory, 2 = minimally satisfactory, 3 = fully successful, 4 = excellent, and 5 = outstanding. NCCHV responders were categorized as top-performing and average-performing responders based on their annual performance review ratings completed by their supervisors (see the “Sampling” section for further details).

All NCCHV calls are audio-recorded, retained for approximately 14 months, and stored on a secure platform for quality improvement purposes. Access to NCCHV audio-recorded calls was obtained for this study. All study procedures were approved by VA’s Central Institutional Review Board (Project #1751884).

### Sampling

NCCHV employed 60–111 responders any given year during fiscal years 2020–2023. Data on a total of 159 unique NCCHV responders and their annual performance ratings from fiscal year 2020 to 2023 were obtained. After 89 responders were excluded because they no longer worked with NCCHV in 2024 at the time data were collected for the study and 3 responders were excluded because they did not have annual performance ratings for fiscal year 2023, there were 67 remaining NCCHV responders. For each responder, their mean annual performance review rating for fiscal year 2022–2023 was calculated. Among all 67 NCCHV responders, the total average performance rating was 4.38 (SD = 0.76) over an average of 2.81 years (SD = 1.28). Responders with a mean performance of 5 were classified as top-performing (*n* = 34) and those with a mean performance rating of less than 5 were classified as average-performing (*n* = 33). The sample of responders was stratified into top and average performers and by the number of years each responder received ratings. Within each stratum, responders were randomly selected for a total of 10 top-performing and 10 average-performing responders.

To maximize follow-up time, the first call sample was drawn for the 20 selected responders from September 2023, and a second and third call were sampled from November 2023 and January 2024, respectively. For each month, the authors created a sampling frame with the upper limit equal to the number of calls received by the responder with the fewest calls. Then, between 1 and the upper limit, a random *n* was chosen and the *n*th call for each responder was selected as long as the call was from a veteran themselves calling as a client (i.e., and not a family member or provider calling on behalf of a veteran client), the contact provided sufficient identification (e.g., full name), and the contact resulted in a consult recorded in the VA system. With three calls per responder and a total of 20 responders, a total of 60 calls were sampled.

### Coding

A codebook of responder behaviors was developed and used to code audio-recorded calls (see codes in Table 1). To develop the codebook, a list of coded behaviors was adapted from a project that assessed the effectiveness of high-performing call center staff tracking viral infection.^15^ These codes were refined for this study to capture NCCHV responder behaviors and to ground them based on empirical literature on active-listening and helpful crisis center call behaviors.^15,16^ The codes were further evaluated after review of NCCHV audio recordings before arriving at a finalized codebook. The final codebook contained nine responder behaviors, including whether (1) the responder is tactful and empathetic, (2) carries on a natural conversation and can adapt to dialogue based on the caller’s responses, (3) is thorough with information gathering needed to determine the caller’s needs and opportunities, (4) emphasizes resources available to the caller, (5) indicates a focus that prioritizes efficiency over rapport, (6) deviates from the NCCHV script, (7) provides inappropriate advice, such as incorrect information, (8) is forceful or rigid in collecting or providing information to callers, and (9) experiences any technology challenges such as poor reception (on the part of caller or responder). For each call, each of these nine responder behaviors was rated dichotomously. If a certain responder behavior was observed to be present, the corresponding code was recorded; if the behavior was absent, the corresponding code was not recorded. Two of the authors (JT and LW) first listened and coded six audio-recorded calls to assess their level of agreement and discuss discrepancies. The coders reached 75% agreement in coding, so the remainder of the audio-recorded calls were split between them for coding. The coders were blinded to the category of the responder (i.e., top-performing vs. average-performing).

**Table 1:** Comparison of mean counts of coded behaviors in the triplicate sample calls of top- and average-performing NCCHV responders

Codes	Top performers (*n* = 10)	Average performers (*n* = 10)	Test of difference *p* value	Effect size difference Cohen’s *d*
**Tactful and empathetic**	**2.0**	**1.2**	**0.10**	**0.77**
Natural conversation/able to adapt	1.8	1.7	0.84	0.09
**Thorough with information gathering**	**2.3**	**1.7**	**0.22**	**0.57**
Emphasizes resources	1.9	1.8	0.84	0.09
Indications of efficiency over rapport	1.1	1.1	1.00	0.00
Deviates from script	0.2	0.4	0.51	−0.30
**Provides inappropriate advice**	**0.0**	**0.1**	**0.33**	**−0.45**
**Forceful/rigid**	**0.2**	**0.6**	**0.30**	**−0.49**
Technology challenges	1.3	1.0	0.42	0.37

Test of differences were based on *t*-tests, and bolded values indicate effect sizes > 0.40

*NCCHV* national call center for homeless veterans

### Data extraction

Coders also extracted self-reported background information on veterans from each of the audio-recorded calls, including identifiers, homeless status, any family obligations, current legal issues, any source of income, and any suicide risk. In addition, other NCCHV administrative data on the immediate outcome of calls as reported by VA homeless staff as well as any previous calls to NCCHV 2 years before or 1 year after calls were also available. These data were linked to three other data sources:

1. VA’s Corporate Data Warehouse (CDW) contained demographics, diagnoses, healthcare use, and clinical encounters with staff in VA homeless programs within 2 years pre- and 1 year post-call to NCCHV. VA outpatient healthcare use included outpatient visits to the emergency department (ED), mental healthcare, primary care, specialty medical care, substance use treatment, and other types of healthcare visits (e.g., a pharmacy visit) and clinical encounters with Veterans Justice Programs and Vocational Rehabilitation. Clinical encounters with VA homeless programs that focus on homelessness and housing instability included Grant and Per Diem (GPD), Health Care for Homeless Veterans (HCHV), and Department of Housing and Urban Development-VA Supportive Housing (HUD-VASH). Inpatient healthcare included stays for medical, mental health, and substance use disorder treatment in the Domiciliary Care for Homeless Veterans program. Diagnoses of pre-existing health conditions, including medical conditions and mental health and substance use disorders, were based on International Classification of Disease, Tenth Revision (ICD-10) codes.
2. VA Homeless Operations Management System (HOMES) contained veterans’ records of participation in VA homeless programs, including GPD, HCHV, HUD-VASH, and two emergency shelter programs, Contract Emergency Residential Services (CERS) and Low Demand Safe Haven (LDSH), 2 years pre- and 90 days post-call.
3. The Homeless Management Information System (HMIS) included veterans’ use of Supportive Services for Veteran Families (SSVF) 2 years pre- and 90 days post-call. More details about veteran homelessness and housing instability data extraction from CDW, HOMES, and HMIS have been described elsewhere.^17^

### Data analysis

To compare behaviors of high-performing and average-performing NCCHV responders, each code was treated as a dichotomous variable (1 = present/0 = absent) in each call. For the total count of times each code was used to characterize a responder’s behavior, the authors calculated the sum across each responder’s three calls. Then, the mean counts of the nine coded behaviors for top-performing and average-performing responders were compared using *t*-tests and effect sizes to see if there was a correlation between the behaviors observed in the call sample and the responders’ annual performance ratings. Baseline characteristics of veterans served by top-performing and average-performing responders were compared using Fisher’s exact, chi-squared, Wilcoxon rank-sum, or *t*-tests and a significance level *α* = 0.05. To compare veterans’ service engagement with VA care 90 days before and 90 days after calls to NCCHV, veterans were stratified by responder group and their healthcare and homeless program usage pre- and post-call were compared using McNemar, Wilcoxon signed-rank, or paired *t*-tests.

## Results

The 10 top-performing responders all received a performance rating from their supervisor of 5.0 (outstanding) for fiscal years 2022–2023 and the 10 average-performing responders received a mean performance rating of 3.5 (SD = 0.6) from their supervisor (between fully successful and excellent). Both groups had ratings for an average of 2.8 years (SD = 1.3). Responders answered between 43 and 379 calls per month with an average of 223.1 calls per month (SD = 83.8) in the timeframe that was sampled. The duration of calls in the sample varied from 4 min and 52 s to 31 min and 54 s with an average duration of approximately 14 min and 12 s (SD = 6 min, 34 s). Among top-performing responders, the average call duration was 14 min, 28 s (SD = 7 min, 10 s) which was not significantly different from average-performing responders, who had an average call duration of 13 min, 55 s (SD = 6 min, 1 s; *p* = 0.75).

### Comparing behaviors of top- and average-performing responders

Table 1 shows the mean number of times each code was used to describe responder behavior in the top- and average-performing responders’ three calls as well as *p* values from *t*-tests and Cohen’s *d* values of the mean ratings for top- and average-performing responders. In the calls of top-performing responders, the codes “tactful and empathetic” (*d* = 0.8) and “thorough with information gathering” (*d* = 0.6) were used more frequently while the codes “provides inappropriate advice” (*d* = −0.4) and “forceful/rigid” (*d* = −0.5) were used less frequently relative to those of average-performing responders.

### Characteristics of veterans served by top- and average-performing responders

Table 2 includes the baseline characteristics of veterans served by the top- and average-performing responders (*n* = 60 veterans). Most callers (58.3%, *n* = 35) reported being at risk of homelessness compared to 41.7% (*n* = 25) experiencing homelessness at the time of their call. Roughly half (51.7%, *n* = 31) of veterans reported that they were accompanied by dependents (e.g., a child, spouse, or pet) and that they received VA disability income (55.0%, *n* = 33). Comparing veterans whose calls were answered by top- and average-performing responders (*n* = 30 veterans in each group), there were no statistically significant differences in these characteristics nor in the proportions of veterans with current legal issues, sources of income, in need of additional VA services, or experiencing suicidality at the time of their calls. Nearly all (*n* = 59) veterans had partial or complete demographic data available in VA records, and most veterans (81.7%, *n* = 49) had previously used VA care before. A substantial portion of veterans had previously contacted NCCHV since 2019 (43.3%, *n* = 26) and had encounters with VA homeless programs (50.0%, *n* = 30) in the 2 years prior to the recorded call that was reviewed for this study.

**Table 2:** Baseline characteristics of veterans served by top- and average-performing NCCHV responders

Baseline characteristics	Total		Top performers (*n* = 30 veterans)		Average performers (*n* = 30 veterans)		Test of difference ***p*** value
Self-reported data							
Current housing situation							
At risk of homelessness	58.3%	35	53.3%	16	63.3%	19	0.43
Homeless	41.7%	25	46.7%	14	36.7%	11	
Accompanied by dependents or has family obligations	51.7%	31	60.0%	18	43.3%	13	0.20
Current legal issues (other than eviction/foreclosure)	26.7%	16	26.7%	8	26.7%	8	1.00
Current sources of income							
VA disability	55.0%	33	50.0%	15	60.0%	18	0.44
Employment	23.3%	14	23.3%	7	23.3%	7	1.00
Other	45.0%	27	46.7%	14	43.3%	13	0.80
None	10.0%	6	6.7%	2	13.3%	4	0.67
In need of additional VA services	21.7%	13	40.0%	7	26.7%	6	1.00
Current suicidality	1.7%	1	0.0%	0	3.3%	1	1.00
*VA administrative data*							
Previously called NCCHV	43.3%	26	46.7%	14	40.0%	12	0.60
Sex							
Female	30.5%	18	34.5%	10	26.7%	8	0.51
Male	69.5%	41	65.5%	19	73.3%	22	
Race^a^							
Black	43.9%	25	39.3%	11	48.3%	14	0.49
White	56.1%	32	60.7%	17	51.7%	15	
Ethnicity							
Hispanic or Latino	17.5%	10	14.3%	4	20.7%	6	0.73
Not Hispanic or Latino	82.5%	47	85.7%	24	79.3%	23	
Any negative discharge	3.4%	2	3.4%	1	3.3%	1	1.00
VA eligibility							
Service-connected disability ≥ 50%	52.5%	31	55.2%	16	50.0%	15	0.70
Service-connected disability < 50%	20.3%	12	24.1%	7	16.7%	5	0.48
No service-connected disability	20.3%	12	17.2%	5	23.3%	7	0.56
Humanitarian emergency	6.8%	4	3.4%	1	10.0%	3	0.61
Homeless programs encounter in past 2 years	50.0%	30	56.7%	17	43.3%	13	0.30
GPD	5.0%	3	3.3%	1	6.7%	2	1.00
HCHV	40.0%	24	43.3%	13	36.7%	11	0.60
HUD-VASH	16.7%	10	20.0%	6	13.3%	4	0.73
Emergency shelter	1.7%	1	0.0%	0	3.3%	1	1.00
SSVF	20.0%	12	26.7%	8	13.3%	4	0.33
Pre-existing conditions							
Mental health disorder	43.3%	26	26.7%	8	60.0%	18	**0.009**
Medical condition	33.3%	20	36.7%	11	30.0%	9	0.58
Substance use disorder	21.7%	13	13.3%	4	30.0%	9	0.21
Outpatient healthcare usage in past 2 years	81.7%	49	76.7%	23	86.7%	26	0.32
ED	46.7%	28	40.0%	12	53.3%	16	0.30
Medical	55.0%	33	53.3%	16	56.7%	17	0.80
Mental health	63.3%	38	50.0%	15	76.7%	23	**0.03**
Primary care	61.7%	37	56.7%	17	66.7%	20	0.43
Substance use	11.7%	7	3.3%	1	20.0%	6	0.10
Other healthcare	51.7%	31	43.3%	13	60.0%	18	0.20
Justice programs	6.7%	4	3.3%	1	10.0%	3	0.61
Vocational rehab	11.7%	7	10.0%	3	13.3%	4	1.00
Inpatient healthcare usage in past 2 years	21.7%	13	16.7%	5	26.7%	8	0.53
Medical	10.0%	6	13.3%	4	6.7%	2	0.67
Mental health	10.0%	6	6.7%	2	13.3%	4	0.67
Substance use	3.3%	2	0.0%	0	6.7%	2	0.49
Domiciliary	8.3%	5	3.3%	1	13.3%	4	0.35
**Baseline characteristics**	**Mean**	**(SD)**	**Mean**	**(SD)**	**Mean**	**(SD)**	***p* value**
*VA administrative data*							
Previous calls to NCCHV	3.4	(2.6)	3.3	(2.5)	3.6	(2.8)	0.79
Days since most recent prior call to NCCHV	167.0	(215.9)	168.2	(216.2)	165.7	(225.1)	0.57
Age	43.1	(14.2)	42.1	(15.1)	44.0	(13.5)	0.62
Count of outpatient healthcare visits in past 2 years							
ED	1.9	(3.9)	1.3	(2.0)	2.5	(5.1)	0.22
Homeless programs (excl. emergency shelter & SSVF)	2.1	(4.0)	2.1	(3.5)	2.1	(4.4)	0.85
Medical	6.3	(9.2)	5.8	(10.0)	6.8	(8.5)	0.52
Mental health	6.9	(11.8)	4.9	(10.4)	9.0	(12.9)	**0.04**
Primary care	3.8	(4.5)	3.8	(4.5)	3.8	(4.5)	0.86
All other types (substance use, other healthcare, justice programs, vocational rehab.)	6.5	(15.3)	5.5	(18.1)	7.5	(12.2)	0.63

*p* values from Fisher’s exact, *χ*^2^ Wilcoxon rank-sum, or *t*-tests

*ED *emergency department, *excl.* excluding, *GPD* Grant and Per-Diem program, *HCHV* Health Care for Homeless Veterans program, *HUD-VASH* Department of Housing and Urban Development (HUD)-Department of Veterans Affairs (VA) Supporting Housing program, *NCCHV* National Call Center for Homeless Veterans, *SSVF* Supportive Services for Veteran Families program, *VA* Department of Veterans Affairs

^a^One Asian veteran served by the average-performing responder group was not included in the *χ*^2^ test given the small cell size

Values in bold indicate *p *< .05

Comparing veterans whose calls were answered by top- and average-performing responders, there were no statistically significant differences in sex, race, ethnicity, discharge status, VA eligibility, prior VA use, or encounters with a homeless program in the 2 years pre-call. However, a higher proportion of veterans who spoke to average-performing responders had a pre-existing mental health disorder (60.0%) than veterans who spoke to top-performing responders (26.7%, *p* = 0.009). Similarly, veterans in the average-performing responder group also had more outpatient mental healthcare visits in the 2 years pre-call (mean of 9.0, SD = 2.4) compared to veterans in the top-performing responder group (mean of 4.9, SD = 10.4, *p* = 0.04). There were no statistically significant differences in other pre-existing health conditions or healthcare usage variables.

### Outcomes of veterans served by top- and average-performing responders

Within 2 weeks after contacting NCCHV, 88.3% of veterans had a documented consult in their VA medical record (83.3% from top-performing and 93.3% from average-performing responders, which was not significantly different). Table 3 shows healthcare and homeless program use for veterans in the 90 days pre- and post-contact to NCCHV stratified by top- and average-performing NCCHV responders. Among veterans served by top-performing responders, the proportion who were engaged with homeless programs increased significantly from 30.0% (*n* = 9) before NCCHV contact to 53.3% (*n* = 16, *p* = 0.04) after NCCHV contact; participation in the HCHV program, specifically, increased significantly from 16.7% (*n* = 5) to 40.0% (*n* = 12, *p* = 0.04). The proportion of veterans in this group who used outpatient VA healthcare also increased significantly from 60.0% (*n* = 18) pre-NCCHV contact to 90.0% (*n* = 27, *p* < 0.01) post-NCCHV contact. Among veterans served by top-performing responders, the mean count of all general healthcare visits, including outpatient healthcare and homeless services (other than emergency shelter and SSVF use), increased significantly from 3.9 (SD = 8.6) pre-NCCHV contact to 12.5 (SD = 21.4, *p* = 0.02) post-NCCHV contact. Among veterans served by average-performing responders, the mean count of all general healthcare visits did not significantly change pre- to post-NCCHV contact (mean of 6.5, SD = 9.2 to mean of 6.8, SD = 10.3, *p* = 0.80).

**Table 3:** Change in veteran healthcare and homeless program use before and after contact with NCCHV, stratified by top- and average-performing NCCHV responders

	Service use outcomes	90 days before NCCHV contact		90 days after NCCHV contact		Test of difference *p *value
Top performers (*n* = 30 veterans)	Homeless programs	30.0%	9	53.3%	16	**0.04**
	GPD	0.0%	0	3.3%	1	1.00
	HCHV	16.7%	5	40.0%	12	**0.04**
	HUD-VASH	13.3%	4	26.7%	8	0.13
	Emergency shelter	0.0%	0	10.0%	3	0.25
	SSVF	13.3%	4	10.0%	3	1.00
	Outpatient healthcare	60.0%	18	90.0%	27	**0.004**
	ED	13.3%	4	16.7%	5	1.00
	Medical	36.7%	11	30.0%	9	0.75
	Mental health	33.3%	10	36.7%	11	1.00
	Primary care	33.3%	10	40.0%	12	0.73
	Substance use	0.0%	0	6.7%	2	0.50
	Other healthcare	13.3%	4	23.3%	7	0.38
	Justice programs	3.3%	1	6.7%	2	1.00
	Vocational rehabilitation	3.3%	1	6.7%	2	1.00
	Inpatient healthcare	3.3%	1	16.7%	5	0.13
	Medical	0.0%	0	6.7%	2	0.50
	Mental health	3.3%	1	6.7%	2	1.00
	Substance use	0.0%	0	0.0%	0	1.00
	Domiciliary	3.3%	1	6.7%	2	1.00
Average performers (*n* = 30 veterans)	Homeless programs	30.0%	9	40.0%	12	0.58
	GPD	0.0%	0	6.7%	2	0.50
	HCHV	16.7%	5	33.3%	10	0.23
	HUD-VASH	6.7%	2	10.0%	3	1.00
	Emergency shelter	0.0%	0	6.7%	2	0.50
	SSVF	13.3%	4	13.3%	4	1.00
	Outpatient healthcare	73.3%	22	90.0%	27	0.18
	ED	26.7%	8	13.3%	4	0.29
	Medical	26.7%	8	36.7%	11	0.45
	Mental health	50.0%	15	53.3%	16	1.00
	Primary care	30.0%	9	26.7%	8	1.00
	Substance use	13.3%	4	13.3%	4	1.00
	Other healthcare	36.7%	11	23.3%	7	0.29
	Justice programs	0.0%	0	3.3%	1	1.00
	Vocational rehabilitation	3.3%	1	6.7%	2	1.00
	Inpatient healthcare usage	10.0%	3	3.3%	1	0.50
	Medical	0.0%	0	0.0%	0	1.00
	Mental health	6.7%	2	0.0%	0	0.50
	Substance use	3.3%	1	3.3%	1	1.00
	Domiciliary	3.3%	1	0.0%	0	1.00

*p* values are based on McNemar’s test

*ED* emergency department, *GPD* grant and per-diem program, *HCHV* health care for homeless veterans program, *HUD-VASH* department of housing and urban development (HUD)-department of veterans affairs (VA) supporting housing program, *NCCHV* national call center for homeless veterans, *SSVF* supportive services for veteran families program

Values in bold indicate *p *< .05

## Discussion

Several observable behaviors differentiated top-performing responders from average-performing responders in a national homeless call center. NCCHV responders were initially categorized based on supervisor ratings of their performance, and the authors were essentially able to validate these ratings by observing actual interactions with veterans who contacted NCCHV and blindly rating their behaviors. Specifically, the top-performing NCCHV responders exhibited more tactfulness and expressed more empathy to veterans who contacted NCCHV than average-performing responders. These findings align with a large body of psychological literature on therapeutic alliance and common factors of psychotherapy that can lead to positive effects,^18,19^ and the present study suggests that this work may also apply to staff at a homeless call center. NCCHV responders and other homeless call center staff may not always be trained in how to deliver services from a therapeutic perspective since their main tasks are to collect data and serve as a dispatch and referral service, but the study findings suggest they may benefit from such training.

Nearly all veterans who contacted NCCHV were referred to VA homeless programs, which is part of the NCCHV service protocol and reported in a previous study.^3^ More notably, veterans who were served by top-performing NCCHV responders showed significant increases in the *utilization*of VA homeless programs and outpatient VA healthcare before and after contact with NCCHV, which was not found among veterans served by average-performing NCCHV responders. A potential explanation is that top-performing NCCHV responders were better at conveying the availability of VA services to veterans that influenced their utilization. Since veterans served by average-performing NCCHV responders had higher baseline rates of mental health diagnoses and mental healthcare use by chance (i.e., random sampling was used), this is also a confounder that may have influenced the findings. Moreover, the observational study design did not allow for any causal inference. But the findings suggest the type of experience that veterans receive by NCCHV (i.e., served by a top- versus average-performing responder) may influence their level of engagement with VA homeless and healthcare services afterwards. In other words, how tactful and empathetic NCCHV responders are may affect how open and willing veterans are to use VA services. It has long been well documented that some veterans have negative perceptions about VA care and are reluctant to seek care at VA facilities.^20–22^ Issues such as institutional distrust and stigma should not be underestimated when helping veterans connect to VA services and the important manner in which NCCHV interacts with veterans. NCCHV does receive calls from veterans who request non-VA resources and decline consults to VA. Further study is needed on this issue and to determine whether they extend to other call centers serving different populations and healthcare systems.

This study had a number of limitations to note. First, a small sample size of 20 NCCHV responders was selected. Due in part to the small sample size, comparison of responder behaviors by *t*-test did not reveal statistically significant differences, whereas comparison using Cohen’s *d* values indicated several substantial differences. Because the veteran sample was larger, tests comparing veteran outcomes by responder type had more statistical power. Veterans were not matched on any baseline characteristics though so those served by average-performing NCCHV responders had more baseline mental health disorders. Replication of these studies with a larger sample size to have statistical power for multivariable analyses is needed. Also, top- and average-performing NCCHV responders were categorized based on supervisor performance reviews, which likely vary by supervisor and may have confounded findings. Finally, there is also great variability in how local VA homeless programs handle NCCHV referrals that may also have affected the results in unmeasured ways and limit generalizability of the study findings. These limitations should be considered in the context of the study’s strengths as well, including implementation of methods to minimize bias (e.g., random sampling of calls, blind ratings), ratings of actual audio-recorded NCCHV calls (not transcripts) that allow for observing behaviors like empathy and tactfulness, and linkage of multiple data sources including healthcare use to allow for analysis of outcomes 3 months after NCCHV contact which is not often available from individuals who contact other call centers.

## Implications for Behavioral Health

Consistent with the psychological literature, the study findings confirm the clinical value of active listening skills, such as being tactful and expressing empathy, in interactions with homeless and at-risk veterans calling a homeless call center. Crisis call center staff would benefit from continued training and feedback on these behaviors, and their audio-recorded calls could be rated as in this study. The study findings may also have broader implications about the importance of “human connection” in an era of proliferating AI agents across many service sectors. Corporations are beginning to use AI in call centers and assistance hotlines,^13,14^ and social service hotlines may follow.^11^ This study suggests there may be an important “human” element to consider in services like homeless call centers that can influence perceived access and engagement, which may in turn affect important psychosocial outcomes like homelessness. Rigorous study is needed on differences between human and AI agents in call centers and careful consideration of any unintended consequences with adoption of AI.

## Conclusion

This study of the VA’s National Call Center for Homeless Veterans found high-performing call center staff, compared to average-performing staff, showed observable differences in tactfulness and empathy in calls with veterans which in turn may have led to increases in engagement with VA homeless and healthcare services for 3 months after their call. These findings may inform future design of call centers as well as the training and evaluation of homeless call center staff so they can best serve clients who are homeless or at-risk of homelessness seeking assistance.

## Data Availability

These data are available upon reasonable request from the corresponding author with proper institutional approvals.

## Declarations

### Reporting Guidelines

This work followed the STROBE guidelines for observational studies.
